# Using data science for medical decision making case: role of gut microbiome in multiple sclerosis

**DOI:** 10.1186/s12911-020-01263-2

**Published:** 2020-10-12

**Authors:** Jasminka Hasic Telalovic, Azra Music

**Affiliations:** University Sarajevo School of Science and Technology, Hrasnicka cesta 3a, Ilidza, 71210 Bosnia and Herzegovina

**Keywords:** Machine learning, Microbiome, Data science, Multiple sclerosis

## Abstract

**Background:**

A decade ago, the advancements in the microbiome data sequencing techniques initiated the development of research of the microbiome and its relationship with the host organism. The development of sophisticated bioinformatics and data science tools for the analysis of large amounts of data followed. Since then, the analyzed gut microbiome data, where microbiome is defined as a network of microorganisms inhabiting the human intestinal system, has been associated with several conditions such as irritable bowel syndrome - IBS, colorectal cancer, diabetes, obesity, and metabolic syndrome, and lately in the study of Parkinson’s and Alzheimer’s diseases as well. This paper aims to provide an understanding of differences between microbial data of individuals who have been diagnosed with multiple sclerosis and those who were not by exploiting data science techniques on publicly available data.

**Methods:**

This study examines the relationship between multiple sclerosis (MS), an autoimmune central nervous system disease, and gut microbial community composition, using the samples acquired by 16s rRNA sequencing technique. We have used three different sets of MS samples sequenced during three independent studies (Jangi et al, Nat Commun 7:1–11, 2016), (Miyake et al, PLoS ONE 10:0137429, 2015), (McDonald et al, Msystems 3:00031–18, 2018) and this approach strengthens our results. Analyzed sequences were from healthy control and MS groups of sequences. The extracted set of statistically significant bacteria from the (Jangi et al, Nat Commun 7:1–11, 2016) dataset samples and their statistically significant predictive functions were used to develop a Random Forest classifier. In total, 8 models based on two criteria: bacteria abundance (at six taxonomic levels) and predictive functions (at two levels), were constructed and evaluated. These include using taxa abundances at different taxonomy levels as well as predictive function analysis at different hierarchical levels of KEGG pathways.

**Results:**

The highest accuracy of the classification model was obtained at the genus level of taxonomy (76.82*%*) and the third hierarchical level of KEGG pathways (70.95*%*). The second dataset’s 18 MS samples (Miyake et al, PLoS ONE 10:0137429, 2015) and 18 self-reported healthy samples from the (McDonald et al, Msystems 3:00031–18, 2018) dataset were used to validate the developed classification model. The significance of this step is to show that the model is not overtrained for a specific dataset but can also be used on other independent datasets. Again, the highest classification model accuracy for both validating datasets combined was obtained at the genus level of taxonomy (70.98*%*) and third hierarchical level of KEGG pathways (67.24*%*). The accuracy of the independent set remained very relevant.

**Conclusions:**

Our results demonstrate that the developed classification model provides a good tool that can be used to suggest the presence or absence of MS condition by collecting and analyzing gut microbiome samples. The accuracy of the model can be further increased by using sequencing methods that allow higher taxa resolution (i.e. shotgun metagenomic sequencing).

## Background

As science is increasingly evolving into a multidisciplinary field, the intersection of several scientific subjects is considered to be the area of potential scientific contribution. The research of the microbiome and the relationship of microbiome with the host organism is one of the scientific fields that demonstrates a swift growth in development [1]. This was enabled by the advancement in data science and bioinformatics tools utilized for the identification and analysis of relevant taxa and its abundances. Additionally, as more samples (both in terms of quantity but also diversity) are sequenced and decoded by bioinformatics tools, the application of data science techniques enables us to gain important insights into medical conditions by examining the data only.

### The human gut microbiome

Microbiota is defined as a network of harmonious and pathogenic microorganisms that are present on and inside the humans. Besides the gut microbiota, the nasal, oral, skin and vaginal microbiota have been examined extensively [2]. The intestinal tract contains the principal mass of human microorganisms. The approximate weight of the microbes present in a human digestive tract is 1.5kg, therefore comprising half of the fecal matter [3].

The association with the human host of majority of microbiota bacteria is either commensal or beneficial, thus considered to be non-pathogenic [4]. Bacteroidetes and Firmicutes are the two most significant phyla bacteria present in the gut microbiota. The number of species-level bacteria present in a single human gut varies. According to [5], a study of 124 individuals presented over 1000 species altogether, and each individual comprised of around 160 distinctive species. From the 160 different species, 18 were present across all subjects, and 75 species were found in most of the subjects. This indicates high variability in abundance of the species. This intersample variance has been a subject of many studies that have inspired the research in this paper.

The makeup of the gut microbiota can be studied using the fecal samples that are collected in a non-invasive manner. Since the methods for collecting samples from other segments of the gastrointestinal system are characterized as invasive, this might suggest that the study of the gut is limited [6]. Nevertheless, the fecal samples distinguish the sufficiently large division in the colon that hosts most of the metabolic activity, and thus, can be used for further analysis [6]. Therefore, the results of the fecal sample analysis are suitable and can provide additional information that is significant to support medical decision making.

### Sequencing techniques: 16s rRNA gene sequencing

The 16s rRNA gene has been used as the key identifier for the classification of microorganisms that reside in the human gut since the mid-1980s [7]. This gene contains conserved and variable regions that enable universal primer construction and facilitate the distinction between different species. The 16s rRNA gene contains approximately 1500 base pairs. The conserved regions of that gene qualify it for amplification and marking in a microbial sample using the PCR technique [8].

### Gut microbiome and medical conditions

The gut microbiota and the human host have a symbiotic relationship. Dysbiosis may occur when commensal bacteria are outnumbered or replaced by pathogenic once. Various intersample variance studies of gut microbiome report potential for utilization of these results for sample classification. The links have been discovered between an imbalance in gut microbiota and various diseases such as colorectal cancer, inflammatory bowel disease (IBD) and irritable bowel syndrome (IBS), diabetes, obesity, metabolic syndrome, malnutrition, and rheumatoid arthritis [9–13]. In the neurological studies, the role of gut microbiota has been identified in Parkinson’s disease and Alzheimer’s disease [14, 15]. The relationship between gut microbes and the development of multiple sclerosis has been explored by recent studies. Multiple sclerosis (MS) is a central nervous system condition that affects humans. The etiology and pathogenesis of MS remain still unknown but dysbiosis has been demonstrated [16]. The discoveries in the field of MS-microbiome association could help discover new ways to identify, treat or prevent the MS relapse.

### Study aim

This study aims to explore the differences between gut microbiome samples (obtained using 16s rRNA sequencing technique) of individuals with and without MS and use those differences to develop a computation model that would likely distinguish those two groups of samples.

## Methods

The paper aims to devise a computational model that will discriminate healthy and MS condition samples. We use data science and machine learning techniques to develop this model. This process starts with intersample analysis and identifications of characteristics of healthy versus MS samples which are based on taxonomical and functional analysis. The discovered variance is used to develop a classification model (based on machine learning techniques) that can be used to identify, with a high probability, if a new sample is with or without MS condition. This approach can be applied to the disorders (other than MS) by building a similar model. This would involve a new intersample analysis and development of a new classification model.

The work in this paper is continuation of the work published in [17]. Besides performing the cross-validation of the previous results, the following extensions have been introduced:

- development of the classification model based on predictive functions,
- validation of the model by testing its classification power on independent sets of gut microbiome data (data even coming from different cultures).

### Dataset

We identified several studies that explored gut microbiome and MS, and therefore potentially have data that could be used in our analysis [18–25]. All the available data were considered and all suitable data from these studies have been included in the datasets that we studied.

In this study we used three datasets. The modelling dataset [18] was used to develop the initial computational model and the samples were taken from the population sampled in the United States (Boston, MA) and reported in 2016. More details about this dataset follow in “Modelling dataset” section.

For validation purposes, a new dataset was constructed. The samples of self-reported healthy individuals were taken from the United States population constructed in the American Gut project [26] and reported in 2018, while MS samples were taken from the Japanese population [19] and reported in 2015. It is interesting to note that the validation dataset had samples coming from different cultures and the only variable that we examined is whether the individuals were diagnosed with MS or not. This approach helps us to validate the computational model for biases that might be introduced by other factors that might significantly influence the gut microbiota such as diet [27]. With the examination of the additional variables, such as age, sex, diet, geography, household microenvironment, we could develop even a more precise computational system. To the best of our knowledge, such a comprehensive dataset, both in terms of metadata available and dataset size needed to support examination of all the interesting variables, does not exist.

#### Modelling dataset

The 16s rRNA sequencing method was used to obtain data sequences analyzed in this study. The first set of samples was obtained by [18]. In the initial multiple sclerosis group, the number of subjects was 60 and in the healthy control group, it was 43. The groups had similar demographic characteristics, with a moderately higher number of males recorded in the MS group. All MS subjects were in relapsing-remitting disease state rather than in active relapse. The demographics of the studied population are shown in Table 1.

**Table 1:** Telalovic and Kilic [17] Dataset [18] description

**Attribute**	**Healthy, N = 43**	**Multiple sclerosis, N = 60**
Age	42.2±9.61	49.7±8.50
Male	6 (14%)	19 (32%)
Female	37 (86%)	41 (68%)
Body mass index	26.4±6.3	27.2±4.7
Caucasian	43	58
Disease Duration	NA	12.8±8.3
Untreated	NA	28

Even though the total number of female and male samples in the dataset is known, the individual samples did not have gender labels. This is very unfortunate as this variable has a great potential in the analysis of MS samples. In the data cleansing phase, the samples of individuals already treated for MS were removed, thus we kept only samples of individuals that received no MS treatment. The number of healthy samples to be used was calculated so the statistical power of the t-test is maximized. For this purpose, the *tt_ind*_*solve*_*power* method from *statsmodels* Python library. The inputs to this function were: effect size (the difference between the two means divided by the standard deviation), alpha value (significance level set to 0.05) and expected power of 80% [28]. Once the optimal size of the control group was calculated, a random subset of available samples was chosen to be put in this group. Unfortunately, the dataset did not have the accompanying metadata (with useful variables such as age/sex/BMI), so the only variable that we had to work with is whether the sample was from MS or control group.

When the data cleaning phase was complete, the final counts were 28 for MS samples and 35 for control.

#### Verification dataset

For verification purposes, we considered two additional datasets. The idea here is to test whether the computational model is dataset specific or it is also successful in classification on additional independent datasets. The 18 samples of individuals with MS disease were obtained from the dataset introduced in [19], and 18 samples of self-reported healthy individuals were obtained from the [26] dataset.

### Taxonomy analysis

The sequences were identified and quantified using the pipeline developed with QIIME2 tools [29]. The pipeline included quality-filtering, denoising and classification steps. The classifier used the *GreenGenes* database at 99% identity (version 13_5). Each identified OTU found in the database, with taxonomy resolution starting from the phylum to the species level, has been assigned a name. Using the database names instead of the OTU identifiers, enables easier data understanding, as the phylogeny of the specific bacteria is identified. The resulting table, containing the sample’s count of each specific bacteria, is outputted from the pipeline. The absolute abundance of bacteria was normalized and used for the intersample variance analysis.

### Predictive functional analysis

Besides bacterial identification, another available information about the gut microbiome is a predictive functional analysis of the present bacteria. In this paper, we explored the usage of this information to identify the intersample variance as well.

To predict the functional composition of a metagenome using 16s marker gene and reference genomes database, the PICRUSt (phylogenetic investigation of communities by reconstruction of unobserved states) computational tool has been used. PICRUSt uses a reconstruction algorithm to predict the gene families present and combines them to compose the whole metagenome. This ‘predictive metagenomic’ approach provides useful insights into the functional links between the phylogeny members [30].

### Random forest classification

Training of the classifier and the development of the classification model was accomplished using the Random Forest (RF) classification algorithm. This decision is based on research reported in [31]. This supervised learning algorithm was constructed using the multiple decision trees generated out of data samples selected randomly [32]. Furthermore, RF algorithm generates a prediction for each tree, picks the optimal solution using voting technique, and calculates an acceptable metrics of the feature importance for model revision. Firstly, the data’s values and targets were separated. Using the *train_test_split* method, the data sets were split into training and testing sets. In our implementation, 80% of data is allocated for training, and 20% is allocated to be used for model testing. The reported scores are averages after a 5-fold cross-validation was performed. We generated 100 decision trees. With the increase in the quantity of decision trees, the classifier’s accuracy was increasing as well, at the expenditure of the computational time. The accuracy of the model was calculated by the comparison between the model’s predicted target data and the actual target data. The identical process was iterated on data from six taxonomic levels and two hierarchical levels of predictive functions separately.

**Fig. 1:**
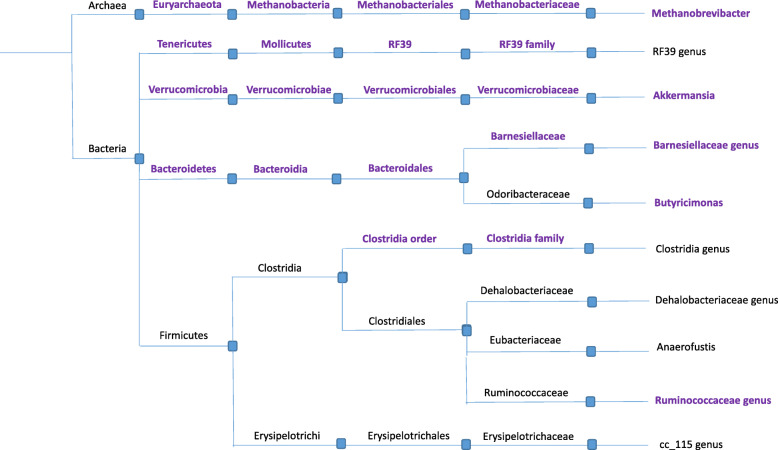
The taxonomy tree includes genus archaebacteria identified as statistically significant at *p*<0.05 along with their phylogenetic tree, used for the development of the classification model. The bolded archaebacteria are the ones that have been identified with high feature importance score by the classifier

The feature importance scores were computed for all the study variables and used for classification model verification. Furthermore, the highest importance features were extracted and applied as the new data for the repeated training the classifier. The accuracy was compared to the initial iteration to ratify that this data forms a robust initial basis for the decision model development. Figure 1 shows the taxonomy of the statistically significant taxa in which taxa with high feature importance scores are emphasized.

## Results

### Accuracy using the model development dataset

#### Taxonomy analysis

As previously stated, we developed a classification model for each of the six taxonomical levels. The pipeline described in “Taxonomy analysis” section established the abundances of bacteria at the six different taxonomic levels. Table 2. summarizes the number of taxa identified for both study groups.

**Table 2:** Telalovic and Kilic [17] Identified bacteria count per taxonomical level in [18] dataset

**Level**	**Healthy control**	**Multiple sclerosis**
Phylum	13	13
Class	29	26
Order	47	39
Family	88	79
Genus	192	174
Species	257	234

In this case, each of these taxa is a potential feature that can be utilized in the classification model for assigning samples to the appropriate groups. The standard procedure that is applied in machine learning is feature selection and its aim is to remove irrelevant and redundant features. The model with the fewer features is more accurate if the most important features are selected. For feature selection we followed approach similar to one described in [33]. In this work, we addressed the feature selection in two steps. Both of the steps improved the accuracy of our model and also reduced the standard deviation of accuracy of different cross-validation runs.

To initially select the classifier’s features, the independent *t-test* was applied and establish the taxa that had a statistically significant difference between the groups. We used *p*<0.05 as the cut-off value for the statistical significance. The identified taxa were the initial candidates for the features. The initial training of the classifier used all the candidate features.

The RF classifier also produces the score for each of the used feature. The feature importance scores obtained from the initial training of the RF classifier were used to further restrict features for the final training of the classifier. The final list of features (taxa), for model training and testing at each taxonomical level, is presented in Table 3. The training and testing data was split using the 5-fold cross validation technique. Multiple runs of the algorithm were run and each time $\frac {1}{5}$ of the data was used for testing and the remaining data was used for training of the model. The average accuracy scores after running 5-fold cross-validation obtained at the taxonomical levels are listed in Table 4. The accuracy reaches 76.82*%* at the genus level of taxonomy which is a significant result.

**Table 3:** Telalovic and Kilic [17] Bacteria with high feature importance score in the classification model using [18] dataset

**Level**	**Name and*p*-value**
Phylum	Euryarchaeota (0.0158), Bacteroidetes (0.0456), Verrucomicrobia (0.0059), Tenericutes (0.0492)
Class	Verrucomicrobiae (0.0059), Bacteroidia (0.0458), Methanobacteria (0.016), Mollicutes (0.0491)
Order	Verrucomicrobiales (0.0059), Bacteroidales (0.0458), Methanobacteriales (0.016), RF39 (0.0482), bacteria from class Clostridia (0.0181)
Family	Verrucomicrobiaceae (0.0059), bacteria from order RF39 (0.0482), Barnesiellaceae (0.0133),
	Methanobacteriaceae (0.016), bacteria from class Clostridia (0.0181), Paraprevotellaceae (0.034)
Genus	Akkermansia (0.0059), bacteria from family Ruminococcaceae (0.0437), Butyricimonas (0.0359),
	bacteria from family Barnesiellaceae (0.0133), Methanobrevibacter (0.0159)
Species	Akkermansia muciniphila (0.0059), bacteria from family Ruminococcaceae (0.0437), bacteria from genus Butyricimonas (0.0359),
	bacteria from family Barnesiellaceae (0.0133), bacteria from genus cc_115 (0.0496)

**Table 4:** Classification accuracy obtained with dataset used for the development of the classification model [18]. The basis for classification was abundance of bacteria on different levels of taxonomy. The discrepancy with [17] is due to the introduction of the cross-validation

**Level**	**Accuracy score**
Phylum	61.90*%*
Class	64.32*%*
Order	69.32*%*
Family	75.16*%*
Genus	76.82*%*
Species	53.44*%*

#### Predictive function analysis

The tables from the PICRUSt analysis containing the KEGG pathways were used as the input for the statistical analysis. Two computational models were developed using the third and second hierarchical levels of KEGG pathways. The feature selection again was done in two steps that resulted in improved accuracy of the model. The first step of the analysis was to identify pathways that were statistically significant between the two groups. For this task, the t-test has been used since the data was normalized in the previous steps.

There were a total of 328 functions related to both, healthy control and MS samples at the third hierarchical level, meaning the lowest level gene function identifiers. All the functions, whose *p*-value is *p*<0.04, were considered to be statistically significant for the classes and extracted to be used in the model training phase. At the third hierarchical level of functions, 91 functions were extracted as statistically significant. The second hierarchical level has also been tested using the t-test. There was a total of 41 functions related to both, healthy control and MS samples. All the functions whose *p*-value is *p*<0.05 were extracted, and the number of those was 20. The list of predictive functions was further restricted by only using the ones with the highest importance as identified by the initial run of the RF algorithm. The final list of the used predictive functions is listed in Table 5.

**Table 5:** Bacteria with high feature importance score in the classification model developed using the dataset from [18]

**Level**	**Predictive functions with high feature importance score**
2nd hierarchical level	Signaling Molecules and Interaction, Amino Acid Metabolism, Excretory System, Lipid Metabolism,
	Genetic Information Processing, Nervous System, Energy Metabolism
3rd hierarchical level	Carotenoid biosynthesis, Influenza A, Glycosyltransferases, Basal transcription factors, Biosynthesis of unsaturated fatty acids,
	Caprolactam degradation, Signal transduction mechanisms, Flavonoid biosynthesis, Caffeine metabolism,
	Chloroalkane and chloroalkene degradation, Non-homologous end-joining, Hepatitis C,
	Chagas disease (American trypanosomiasis), Butirosin and neomycin biosynthesis, Chlorocyclohexane and chlorobenzene
	degradation, Phenylalanine, tyrosine and tryptophan biosynthesis, Ubiquinone and other terpenoid-quinone biosynthesis,
	Vibrio cholerae infection, Nitrotoluene degradation, Steroid hormone biosynthesis, Aminoacyl-tRNA biosynthesis, Steroid
	biosynthesis, Bacterial toxins, Novobiocin biosynthesis, Phenylalanine metabolism, Pantothenate and CoA biosynthesis,
	Meiosis – yeast, Cell cycle

The functions identified as significant were used for the development of additional two predictive models (one using second and the other third hierarchical level of the KEGG pathways). The training and testing data was split using the 5-fold cross validation technique. Multiple runs of the algorithms were run and each time $\frac {1}{5}$ of the data was used for testing and the remaining data was used for training of the model. The average accuracy scores obtained at the two hierarchical levels are listed in Table 6.

**Table 6:** Classification accuracy obtained with dataset used for the development of the classification model [18]. The basis for classification was different hierarchical levels of KEGG pathways

**Level**	**Accuracy score**
2nd hierarchical level	62.03*%*
3rd hierarchical level	70.95*%*

### Testing the classification model on independent datasets

The developed model was tested using two independent datasets. The first dataset [19] provided the 18 samples of individuals with the MS disease. The second dataset [26] provided samples of 18 individuals that self-reported as healthy.

**Fig. 2:**
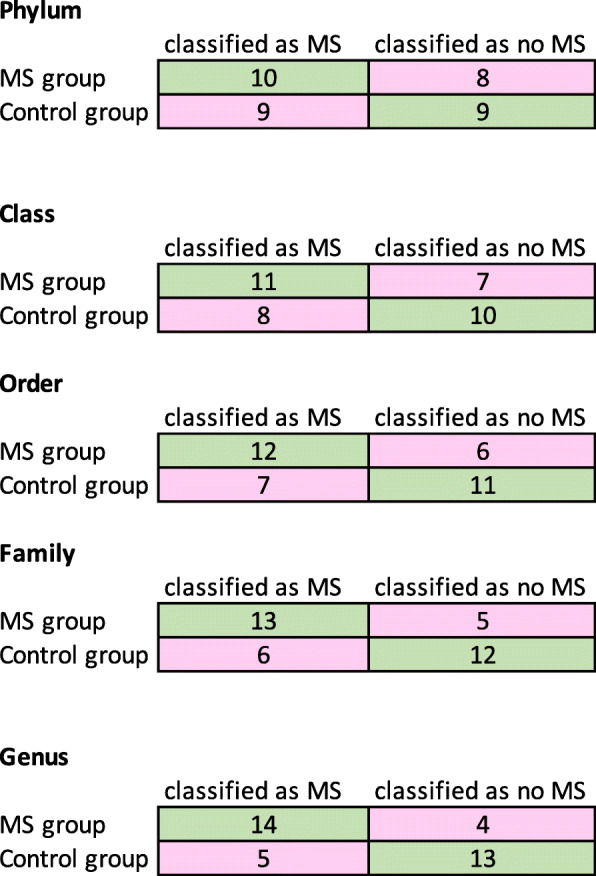
The confusion matrix visualises the number of samples that were correctly (green background) and incorrectly (pink background) classified. The higher the taxonomy resolution, the more accurate classification results were obtained

The combined accuracy of prediction using the abundance of bacteria on different taxonomy levels is summarized in Table 7. Table 8 contains accuracies of the model predicting MS samples and Table 9 contains accuracies of predicting non-MS samples. The accuracy for predictive based on predictive functions the accuracy is summarized in Table 10. In Fig. 2 the confusion matrix visualizes how the number of correct classifications (in both MS and control groups) for classifiers that used taxa at a higher resolution as features. The diagonal values are the ones that were correctly classified.

**Table 7:** Classification accuracy obtained with validation datasets [19, 26]. The basis for classification was abundance of bacteria on different levels of taxonomy

**Level**	**Accuracy score**
Phylum	52.78*%*
Class	58.33*%*
Order	63.89*%*
Family	69.44*%*
Genus	75%
Species	51.56*%*

**Table 8:** Classification accuracy obtained with validation datasets [19]. The basis for classification was abundance of bacteria on different levels of taxonomy. That dataset contains samples of individuals with MS disease

**Level**	**Accuracy score**
Phylum	55.56*%*
Class	61.11*%*
Order	66.67*%*
Family	72.22*%*
Genus	77.78*%*

**Table 9:** Classification accuracy obtained with validation datasets [26]. The basis for classification was abundance of bacteria on different levels of taxonomy. The dataset contains samples from individuals that self reported as healthy

**Level**	**Accuracy score**
Phylum	50.00*%*
Class	55.56*%*
Order	61.11*%*
Family	66.67*%*
Genus	72.22*%*

**Table 10:** Classification accuracy obtained with validation datasets [19, 26]. The basis for classification was different hierarchical levels of KEGG pathways

**Level**	**Accuracy score**
2nd hierarchical level	58.83*%*
3rd hierarchical level	67.24*%*

Even though our two validation datasets come from two different studies, the classification accuracy and its trends are very similar. This data demonstrates that classifier performance is robust to the batch effect.

## Discussion

The purpose of this study was to study gut microbiota bacterial diversity of MS patients and develop a computational model to distinguish MS patients from the healthy patients by examining their gut microbiome sample 16s amplicon sequences. We developed eight such classifiers and validated their accuracy on an independent dataset.

To develop classifiers, we needed to first identify the important features - those are the taxa (or predictive metabolic functions) that had a significant difference between the MS and control group. The comparison between our results and previous MS studies is summarized in Tables 11 and 12. It can be seen that our computations mostly agree with previous findings (except in one case). Also, our computation provides additional statistically significant bacteria that can be use as classification features and we also provide bacteria at additional taxonomy levels (we could not find any studies that examined the class or order level of bacteria taxonomy). The value of the data science approach is that we were not able only to identify important features, but we also give a single prediction, based on the values of all the important features, that classifies a sample into two groups.

**Table 11:** Comparison of results of this study with other MS studies; *↑* indicates that MS samples have statistically significant increase in abundance of a bacteria and *↓* indicates that MS samples have statistically significant decrease in abundance of a bacteria (* indicates that results are not statistically significant); green color indicates agreement of our results and other MS studies; orange color indicated disagreement of our results and previous MS studies; when our results are in black color, we did not have an MS study to compare those results with

**Level**	**Bacterium**	**This study**	**[**19**]**	**[**22**]**	**[**18**]**	**[**20**]**	**[**21**]**	**[**23**]**	**[**24**]**
Phylum	Euryarchaeota	*green↑*			*↑*	*↑*			
Phylum	Bacteroidetes	*↓*							
Phylum	Verrucomicrobia	*green↑*			*↑*				
Phylum	Tenericutes	*↓*							
Phylum	Firmicutes					*↑*			
Phylum	Actinobacteria		*↑*						*↑*
Phylum	Proteobacteria								*↑*
Phylum	Fusobacteria					*↓*			
Family	Methanobacteriaceae	*↑*							
Family	Verrucomicrobiaceae	*↑*							
Family	uncultured (Costridium)	*↑*							
Family	Barnesiellaceae	*↓*							
Family	Paraprevotellaceae	*↓*							
Family	Uncultured (RF39)	*↓*							
Family	Lachnospiraceae					*↓*			
Family	Bacteroidaceae						*↓*		
Genus	Methanobrevibacter	*green↑*			*↑*				
Genus	Desulfovibrio	*green↑*				*↑*			
Genus	Anaerofustis	*↑*							
Genus	Akkermansia	*green↑*		*↑*	*↑*				
Genus	Butyricimonas	*green↓*			*↓*				
Genus	Uncultured (Ruminococcaceae)	*green↓*				*↓*			
Genus	Uncultured (RF39)	*↓*							
Genus	Ruminococcus	*green↑*^∗^					*↑*		
Genus	Bifidobacterium	*green↑*^∗^	*↑*						
Genus	Faecalibacterium	*green↓*^∗^	*↓*				*↓*		
Genus	Prevotella	*green↓*^∗^	*↓*					*↓*	
Genus	Streptococcus	*orange↓*^∗^	*↑*						
Genus	Acinetobacter			*↑*					
Genus	Parabacteroides			*↓*				*↓*	
Genus	Bilophila					*↑*			
Genus	Christensenellaceae					*↑*			
Genus	Bacteroides		*↓*						
Genus	Anaerostipes		*↓*						
Genus	Pseudomonas							*↑*	
Genus	Mycoplana							*↑*	
Genus	Haemophilus							*↑*	
Genus	Dorea							*↑*	
Species	Methanobrevibacter smithii	*green↑*		*↑*					
Species	Akkermansia muciniphila	*green↑*		*↑*					
Species	Butyricimonas virosa	*green↓*		*↓*

**Table 12:** Comparison of results with other MS studies; *↑* indicates that MS samples have statistically significant increase in predictive metabolic function and *↓* indicates that MS samples have statistically significant decrease in predictive metabolic function; green color indicates agreement of our results and other MS studies; we did not have an MS study to compare with our results in black color

**Level**	**Predictive metabolic function**	**This study**	[25]
**2nd**	**Signalling molecules and interactions**	***↓***	
2nd	Energy metabolic functions	*↓*	
2nd	Excretory system functions	*green↓*	*↓*
2nd	Signal transduction mechanisms	*↓*	
2nd	Replication and repair functions	*↓*	
2nd	Amino acid metabolism	*green↑*	*↑*
2nd	lipid metabolism	*↑*	
2nd	Inorganic ion transport and metabolism	*↑*	
2nd	Unknown functions	*↑*	
3rd	Chromosome functions	*↓*	
3rd	Peptidases functions	*↓*	
3rd	Homologous recombination functions	*green↓*	*↓*
3rd	DNA replication	*↓*	
3rd	Peroxisome and cyan amino acid metabolism	*↓*	
3rd	Vitamin B6 metabolism	*green↓*	*↓*
3rd	*β*-alaine metabolism	*↓*	
3rd	Inorganic ion transport and metabolism	*↓*	
3rd	Mismatch repair functions	*↓*	
3rd	Galactose metabolism	*↓*	
3rd	Steroid hormone biosynthesis	*green↑*	*↑*
3rd	Tuberculosis functions	*↑*	
3rd	Bacterial secretion system	*↑*	
3rd	Influenza A	*↑*	
3rd	Valine, leucine and isoleucine biosynthesis	*↑*	
3rd	Hepatitis C	*↑*	
3rd	Cell motility and secretion	*↑*	

The classifiers based on the abundance of bacteria are more accurate than ones based on predictive functions. We need to be careful about drawing conclusions here as these results do not suggest that there exist less variance in the metabolic potential than in microbiome structure. The tool that we used (PICRUSt), uses microbiome structure to predict the metabolic potential and has limited accuracy. To fully understand the potential of the metabolic influence, we would need to use metabolomics.

The accuracy rises as we look at the higher resolution of taxonomy. As we used samples obtained using the 16s rRNA sequencing technique, we expected the abundance to be accurate up to the genus level as this the limitation of this technique described in the literature [34]. This is because related bacterial species may have almost identical 16s rRNA gene sequences which makes it hard to distinguish them in the bioinformatics pipelines. We indeed observed that our accuracy started to drop at the species level which confirms the previous findings (Tables 4 and 7).

The 16s rRNA sequencing technique distinguishes MS patients from healthy ones with over 70% accuracy using a test considered to be non-invasive. This value represents a significant value for medical decision making. Though this is a valuable contribution, further improvements can be made so that accuracy would be raise.

Overall, this study considered 99 samples, which is a somewhat small number for data science. The mitigating factors are: only two groups were studied, classification was binary, and the sizes of the groups were approximately the same. More samples need to be considered to construct even more applicable and accurate results. The usage of the independent datasets for the validation of the model(s) strengthens our results greatly. With an independent dataset, we were able to achieve similar classification power and that confirms the relevance of the classification model amidst the sample size used. The availability of more samples of individuals with MS condition, as well as improvements in sequencing resolution, would possibly allow the creation of an even more accurate classification model(s).

The human gut contains several sections that host microbial communities but exhibit different environments. The only non-invasive method accessible for collecting gut samples is via stool. Determining the bacterial origin and contributions of different sections of the gastrointestinal system can be difficult. Besides, there are many parameters that can influence intrasample variance such as different age, sex, ethnicity, genetic backgrounds, different diet habits, and life in different environments [12]. In future work, the availability of samples with such metadata would enable us to remove the variance that is introduced by parameters other than the studied condition.

The change of sample sequencing technique can introduce further improvements. The candidate technique is shotgun metagenomics sequencing. As this technique reports accuracy at the finer resolution of taxonomy than 16s, we would be able to continue accuracy improvement by using the models developed on a taxonomic level beyond the genus [35]. This would generate even more reliable results. The shotgun metagenomic sequences continue to be expensive to obtain, and thus 16s data is more easily available. The solid investigation results can shape a solid reason for creating models that can clarify the structure and capacity of the microbial network, and possibly give further knowledge into the connection between microbiome and infection states.

The findings presented here may be used to construct models that would distinguish medical conditions other than MS (i.e. Parkinson’s and Alzheimer’s disease).

To apply the methods presented in this paper in other clinical domains, we would need to perform the following steps:

- obtain a significantly sized set of samples for both condition and control groups,
- perform bioinformatics algorithms to extract desired information from samples (either bacteria abundance or predictive metabolic function),
- perform statistical analysis of obtained data to get a set of distinguishing features to provide as an input for the training of a classifier,
- train the classifier and use the results to conclude the list of features,
- use classifier to predict that an unknown sample is either with the condition or not.

## Conclusions

This paper describes the process of developing a computational model that discriminates microbiome samples of healthy individuals and ones diagnosed with MS. In addition to performing intersample analysis based on taxa abundances, the intersample analysis of predictive functions in the human organism was also performed. The initial development of this work was started in [17]. In this paper, in addition to the more accurate specification and evaluation of the initial model based on taxa, we also presented a model based on the predictive functional analysis. Another major contribution is the evaluation of all the developed models for accuracy on samples that came from the independent dataset(s).

Several previous studies report similar findings on significant taxa for MS [18–23]. The research question had the aim to determine if the taxonomic and functional gut bacteria diversity is a significant factor that can be utilized to develop a machine learning classifier that will distinguish multiple sclerosis samples from the control ones. We developed such a classifier, and it demonstrated a significant accuracy (around 70%).

While we demonstrated that a classifier can distinguish MS and healthy samples, we did not include samples of other cohorts in our study (i.e. other medical conditions) so we have not evaluated how those cohorts would be classified in our model. For this reason, further studies in this direction are needed. In addition to increasing the accuracy, additional cohorts need to be studied. The results of this approach should be taken with caution and augmented with other diagnostics, especially for individuals who do not clearly belong to the two studied groups.

We were limited by the dataset(s) that are currently available. We were not able to examine important variables that we have evidence that either affect MS condition or microbiome structure. Those variables include but are not limited to: age, sex, diet, BMI, geography, household microenvironment... In order to analyze these variables, we need datasets with the appropriate metadata and significant size for multivariable analysis. The availability of such datasets in the future could allow creation of even more precise computational models.

## Data Availability

The datasets analyzed during the current study are available at the following repositories: 1. European Nucleotide Archive https://www.ebi.ac.uk/ena/data/view/PRJNA321051Accession numbers: MS samples (28) - SRX1759626, SRX1759627, SRX1759630, SRX1759632, SRX1759633, SRX1759641, SRX1759642, SRX1759653, SRX1759669 - SRX1759676, SRX1759697 - SRX1759699, SRX1759701 - SRX1759708, SRX1759719 Control/healthy samples (35) - SRX1759621 - SRX1759625, SRX1759654 - SRX1759668, SRX1759682 - SRX1759696 2. European Nucleotide Archive https://www.ebi.ac.uk/ena/data/view/PRJEB11419Accession numbers: ERR4019675 - ERR4019684, ERR4020397 - ERR4020401, ERR4020423 - ERR4020425 3. Bioinformation and DDBJ https://trace.ddbj.nig.ac.jp/DRASearch/experiment?acc=DRX002006Accession numbers: DRA000672, DRA000673, DRA000675, DRA000676, DRA000678-DRA000684, DRA002866-DRA002874.

## Abbreviations

KEGG: Kyoto encyclopedia of genes and genomes
MS: Multiple sclerosis
OTU: Operational taxonomic unit
PCR: Polymerase chain reaction
PICRUSt: Phylogenetic investigation of communities by reconstruction of unobserved states
QIIME: Quantitative insights in microbial ecology
RF: Random forest (classifier)
rRNA: Ribosomal ribonucleic acid
